# The common murine retroviral integration site activating *Hhex* marks a distal regulatory enhancer co-opted in human early T-cell precursor leukemia

**DOI:** 10.1016/j.jbc.2025.108233

**Published:** 2025-01-27

**Authors:** Joyce Hardwick, Javier Rodriguez-Hernaez, Giovanni Gambi, Bryan J. Venters, Yan Guo, Liqi Li, Paul E. Love, Neal G. Copeland, Nancy A. Jenkins, Dimitrios Papaioannou, Iannis Aifantis, Aristotelis Tsirigos, Mircea Ivan, Utpal P. Davé

**Affiliations:** 1 Division of Hematology/Oncology, Indiana University School of Medicine, Indianapolis, Indiana, USA; 2 Department of Medicine, R.L. Roudebush Indianapolis VA Medical Center, Indianapolis, Indiana, USA; 3 Division of Precision Medicine, New York University Genome Center, New York, New York, USA; 4 Department of Pathology, New York University Grossman School of Medicine, New York, New York, USA; 5 Department of Molecular Physiology and Biophysics, Vanderbilt University Medical Center, Nashville, Tennessee, USA; 6 Epicypher, Inc, Durham, North Carolina, USA; 7 Department of Public Health Sciences, University of Miami, Miami, Florida, USA; 8 Section on Hematopoiesis and Lymphocyte Biology, NICHD, NIH, Bethesda, Maryland, USA; 9 Department of Genetics, M.D. Anderson Cancer Center, Houston, Texas, USA; 10 IU Simon Comprehensive Cancer Center, Indianapolis, Indiana, USA

**Keywords:** transcription, mutagenesis, leukemia, retrovirus, chromatin, LMO2, LDB1, complexes, promoter, enhancer, looping

## Abstract

The *Hhex* gene encodes a transcription factor that is important for both embryonic and post-natal development, especially of hematopoietic tissues. *Hhex* is one of the most common sites of retroviral integration in mouse models. We found the most common integrations in AKXD (recombinant inbred strains) T-ALLs occur 57-61kb 3′ of *Hhex* and activate *Hhex* gene expression. The genomic region of murine leukemia virus (MLV) integrations has features of a developmental stage-specific cis regulatory element (CRE), as evidenced by ATAC-seq in murine progenitor cells and high H3K27 acetylation at the syntenic CRE in human hematopoietic cell lines. With ChIP-exonuclease, we describe occupancy of LIM domain binding protein 1 (LDB1), the constitutive partner of the LIM Only-2 (LMO2), GATA1, and TAL1 transcription factors at GATA sites and at a composite GATA-E box within the CRE. With virtual 4C analysis, we observed looping between this +65kb CRE and the proximal intron one enhancer of *HHEX* in primary human ETP-ALLs and in normal progenitor cells. Our results show that retroviral integrations at intergenic sites can mark and take advantage of CREs. Specifically, in the case of *HHEX* activation, this newly described +65kb CRE is co-opted in the pathogenesis of ETP-ALL by the LMO2/LDB1 complex.

Retroviral insertional mutagenesis (RIM) has been a highly informative genetic screen for cancer gene identification and an adverse effect observed in gene therapy (1, 2, 3, 4, 5). Many *bona fide* human oncogenes and tumor suppressor genes were initially implicated through RIM studies with endogenous murine leukemia virus (MLV) in related recombinant strains (6). In RIM studies, MLV proviral integration sites are readily cloned and mapped from murine leukemias and lymphomas (2). MLV integrations occur semi-randomly with a preference for the 5′ end of transcriptional start sites (7). Thus, deregulated genes are usually near MLV integrations, but integrations have also been observed quite distant from genes of interest (8, 9). One prominent example is the unique clustering of MLV integrations in the gene neighborhood of the *Hematopoietically-expressed homeobox (Hhex)* gene (10).

*Hhex* is a non-clustered homeobox transcription factor important in embryonic development and in post-natal maintenance of liver, thyroid, and hematopoietic tissues and the second most common gene to be insertionally mutated in AKXD screens (3). Conditional knockout mouse models have shown that *Hhex* has a role in the cycling of hematopoietic stem cells and in the development of common lymphoid progenitor cells (11, 12). Genetic screens in AKXD mice implicate *Hhex* as an oncogene. There is also human data in support of this. In AML, *HHEX* is part of a rare chromosomal translocation, *NUP98-HHEX* (13). We previously found that *HHEX* is upregulated in human T-ALL and in the *CD2-Lmo2* transgenic mouse model of T-ALL, mainly in the immature Early T-cell precursor subtype (14). Based on these results and the idea that *LIM domain Only-2 (Lmo2)* and *Hhex* are mutually exclusive integrations in AKXD models, we found that *Hhex* is a downstream target of *Lmo2* in T-ALL (14). *Lmo2* is insertionally mutated in RIM models and in gene therapy-induced T-ALLs (1, 2). *Lmo2* encodes a small protein with two LIM domains that is constitutively bound to LIM domain binding protein 1 (Ldb1) (15). Lmo2/Ldb1 is responsible for scaffolding transcription factors such as class II basic helix-loop-helix proteins TAL1 and LYL1 and GATA factors into the LMO2/LDB1 multisubunit complexes at promoters and enhancers of critical developmental genes (15, 16, 17, 18). LDB1 and other subunits of this complex were bound to an enhancer in intron one of *Hhex* (14). In this study, our data on the frequent integrations downstream of *Hhex* converged with our investigation of *HHEX* activation by the LMO2/LDB1 complex. We present evidence that the cluster of integrations of MLV far downstream of *Hhex* marks a cis-regulatory element (CRE) that shows developmental stage-specific chromatin accessibility and is bound by the LMO2/LDB1 complex. This newly identified CRE loops to the proximal promoter of *HHEX* to activate the gene in ETP-ALLs. These data have implications for interpreting retroviral integrations, for the activation of oncogenes, and for *LMO2*-driven ETP-ALL.

## Results

### Common retroviral integrations activating *Hhex* expression are at open chromatin regions

**Figure 1:**
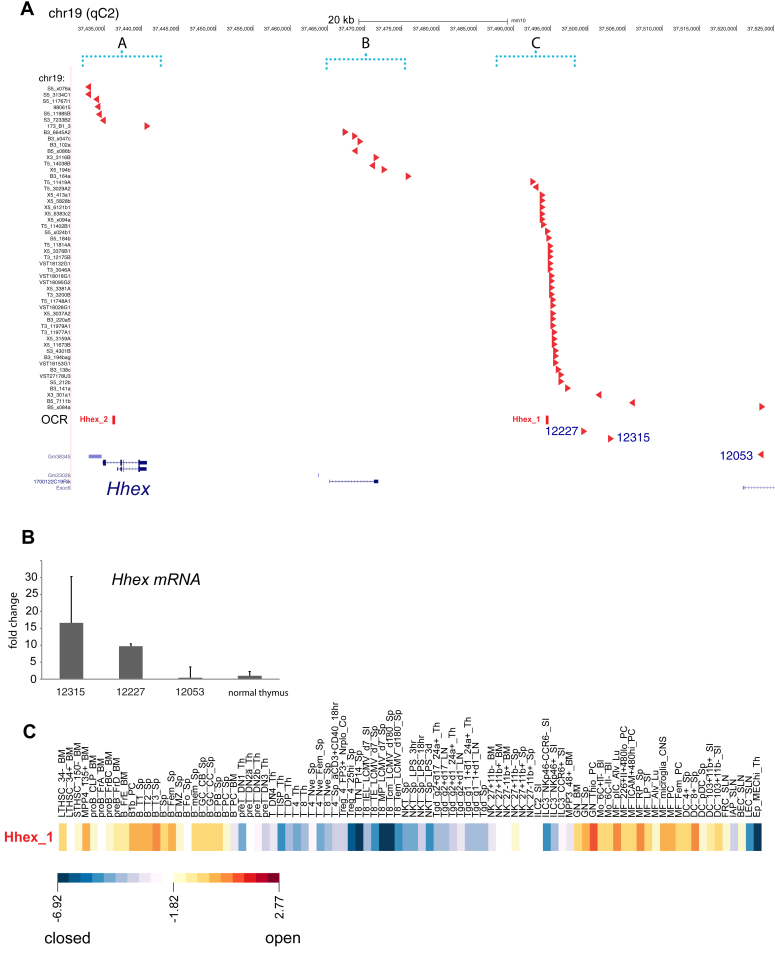
**Retroviral integration sites cluster in three groups near the *Hhex* gene.***A*, schematic shows a genomic window encompassing the *Hhex* gene on murine chromosome 19 and retroviral integrations in its vicinity, divided into three clusters (*red arrows* denoting orientation with respect to the *Hhex* TSS) cloned from AKXD models. Retroviral integrations and their neighboring genomic sequences are listed in Table S1. The OCR row denotes open chromatin regions (Hhex_1 and Hhex_2 in red) from ATAC-seq results further shown in 1C and listed in Figure S1 and Table S2. *B*, bar graph showing qRT-PCR analysis of *Hhex* mRNA in murine T-ALLs (12227, 12315, 12053) normalized to *Hhex* mRNA abundance in normal thymic RNA (fold change above thymus levels is shown). *C*, heat map shows ATAC-seq reads in various normal hematopoietic cell populations at genomic region Hhex_1 shown in 1A. The scale shown below represents the log_2_(value/value_row_mean_) in the color scale bar; *red* and *yellow bars* are open chromatin regions whereas *blue* bars show closed chromatin; the log-transformed values are listed in Table S2.

*Hhex* is the second most common gene to be insertionally mutated in murine retroviral insertional mutagenesis screens (3, 4, 19). All of the murine models with *Hhex* integrations developed lymphoid neoplasms, most commonly T-cell acute lymphoblastic lymphomas (T-ALLs), according to updated genetic and immunophenotyping analyses (2, 20, 21). Cloned retroviral integrations from these tumors mapped to three common insertion site (CIS) clusters: (A) 5′ of the start site (n = 5); (B) +32-40kb (n = 8); and, (C) +57-61kb (n = 33) (Fig. 1 and Table S1). The common integrations at cluster C (defined by the addresses of the most 5′ and 3′ RIS in mm10: chr19:37492367–37495953) were all in the same orientation to the *Hhex* gene and mapped to within this 3586 bp region but six retroviral integrations occurred outside of this window, 3′ of *Hhex*. To better explain the impact of cluster C on gene expression, we analyzed RNA that was available from 3 AKXD21 T-ALLs (12315, 12227, 12053) by qRT-PCR for *Hhex* transcripts. T-ALLs 12227 and 12315 with integrations within cluster C showed 9-15-fold upregulation of *Hhex* mRNA compared to normal thymus whereas T-ALL 12053 showed no upregulation (Fig. 1*B*). Thus, retroviral integrations within cluster C were associated with *Hhex* activation whereas integration outside of this cluster did not show *Hhex* upregulation (10). The 3.586kb region of frequent integration showed conservation across multiple vertebrate species. We analyzed ATAC-seq data performed in normal hematopoietic cell populations (22). The two highest-scoring open chromatin regions (OCR) as determined by ATAC-seq were within cluster C and *Hhex* intron 1 (Figs. 1, *A* and *C*, and S1). We identified (OCR-1; chr19: 37495419–37495470) as a region of chromatin accessibility in specific developmental stages with maximal ATAC-seq tags in the most immature hematopoietic stem and progenitor cell populations, B-cell progenitor cells, and myeloid progenitor cells but not in T cells (Figs. 1*C*, S1, Table S2) (14, 23, 24). Although *Hhex* intron one was previously shown to contain an enhancer, these ATAC-seq data implicate the 3.586kb cluster C as a previously unrecognized cis-regulatory element (CRE).

### Human genome regions syntenic to cluster C are bound by LDB1 in human ETP-ALL

**Figure 2:**
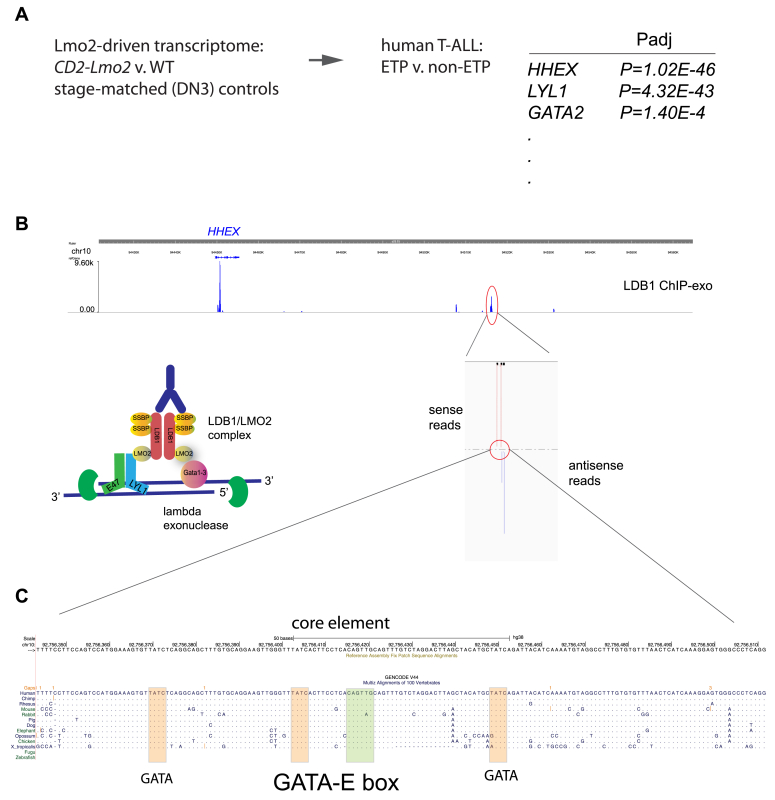
**LDB1 binds to the genomic region syntenic to cluster C in human ETP-ALL cells.***A*, the Lmo2-driven transcriptome was defined as those genes differentially expressed in T-cell progenitor cells from *CD2-Lmo2* transgenic mice. We searched for overlap between these putative Lmo2-driven transcripts and genes differentially expressed in human ETP v. non-ETP-ALL, generating a list of 382 genes; abbreviated table shows some prominent genes and their adjusted *p* values with the full gene list available in Table S3. *B*, genome browser window (hg19) shows ChIP-exonuclease (−exo) peaks from LOUCY T-ALL cells at *HHEX* intron one and at +65kb from the *HHEX* TSS. Reads from sense and antisense strands are shown below the peak at +65 Kb; schematic on *left* shows the LMO2/LDB1 complex and the ChIP-exo technique; *right*. The overlap of sense and antisense strands are regions of occupancy of the LDB1 protein. *C*, shows a zoomed in genomic sequence within the LDB1 ChIP-exo peak annotated for GATA (*orange*) and E box (*green*) sites and their conservation across vertebrate species.

The common integrations at cluster C suggest an oncogenic role for *HHEX* in T-ALL. *HHEX* is transcriptionally upregulated in the Early T-cell Precursor (ETP) subtype of T-ALL in multiple gene expression studies of human T-ALL (14, 25, 26, 27). Both the murine and human ENSEMBL databases show long and short mRNA isoforms with alternate first exons (Human: ENST00000472590.6 and ENST00000282728.10). We analyzed human hematopoietic cell lines and three primary Early T-cell Precursor ALL samples for isoform expression. We observed the long isoform as the predominantly expressed *HHEX* transcript including the ETP-ALL cell lines models, LOUCY and CUTLL3 (see Fig. S2). Forced expression of the long isoform cDNA has been shown to induce T-ALL in mouse models. *HHEX* expression is concordant with *LMO2* in the ETP-ALL subtype of T-ALLs (27). To probe whether the ETP transcriptome was regulated by *LMO2*, we defined *Lmo2*-driven transcripts as differentially expressed between murine *CD2-Lmo2* transgenic T-cell progenitors (DN3a and DN3b) *versus* approximate developmental stage-matched (DN3a) wild type T-cell progenitors (28). This gene list of putative Lmo2-driven transcripts was significantly enriched in the human ETP-ALL transcriptome (Early T-cell Precursor ALL v. non-ETP T-ALL, *p* < 0.0001, see Table S3) (14); 382 genes were present in both gene lists with expression changes in the same direction, accounting for 27% of the differentially expressed genes in ETP-ALL (cutoff of P_adj_ < 0.05, see Table S4). *HHEX*, *LYL1*, and *GATA2* were in the Lmo2-driven ETP-ALL transcriptome and significantly enriched in the ETP-ALL subtype (Fig. 2*A* and Table S4) in our studies and in multiple other transcriptomic analyses of human T-ALL (14, 25, 26, 27, 29). Multiple lines of evidence have shown that *HHEX* is a downstream target of *LMO2* in human T-ALL (14). For example, in murine retroviral models, *Lmo2* and *Hhex* were mutually exclusive integrations (14); and, we previously showed occupancy of LMO2 and its partner, LDB1 at the intron one enhancer of *HHEX* in multiple human leukemic cell lines but it was not clear whether this enhancer was sufficient to activate *HHEX* in ETP-ALL. Furthermore, data from immunoprecipitations, EMSA, and gel chromatography of ETP-ALL nuclear extracts suggest that LMO2 and LDB1 are constitutive partners (15, 18). Since LMO2 is difficult to capture in ChIP assays (30), we performed a detailed chromatin immunoprecipitation-exonuclease (ChIP-exo) analysis of LDB1 occupancy throughout the genome of LOUCY cells, a human cell line model of ETP-ALL with the transcriptomic signature shown in Figure 2, *A* and *B*. We found that intron one of *HHEX* was the highest LDB1 ChIP-exo peak across the whole LOUCY genome. Additionally, we discovered a strong peak of LDB1 occupancy at +65kb 3′ of *HHEX*, the exact genomic region syntenic to cluster C shown in Figure 1. The ChIP-exo technique allowed us to narrow the region of occupancy to a core element that was highly conserved amongst multiple vertebrate species (Fig. 2*B*) (31). This core element contained a composite E box-GATA motif preferred by the LMO2/LDB1 protein complex (17, 18) which was flanked by multiple lone GATA sites also within peaks of LDB1 occupancy based on the ChIP-exo reads (Fig. 2*C*) (28, 32).

### +65kb element and its properties

**Figure 3:**
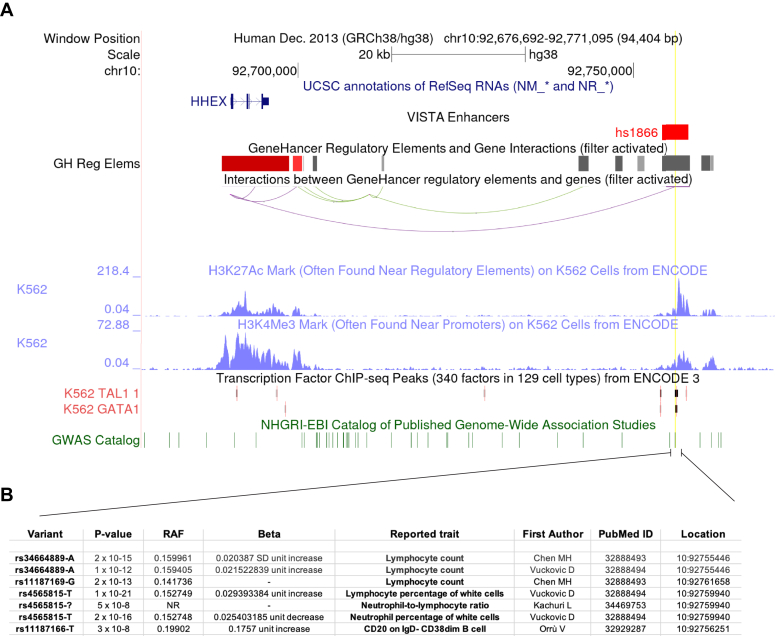
**The human +65kb cis-regulatory element (CRE) and its properties.***A*, genome window from human chromosome 10 is shown syntenic to the murine *Hhex* locus shown in Figure 1*A*. *Vertical green lines* show human single nucleotide polymorphisms from the NHGRI-EBI catalog. The sequence shown in Figure 1*D* was BLAT aligned to the human genome and is shown as a *yellow vertical line*. Gene tracks below show that this region is a candidate enhancer in GeneHancer (with evidence from ENCODE project Z-lab Enhancer-like regions, Ensembl regulatory build, FANTOM5, and VISTA (labeled as hs1866). Putative interactions with the *HHEX* gene are shown as curves to the TSS. Below these curves are the *blue* peaks showing H3K4me3 and H3K27ac from K562 cells. Below this, are shown areas of TAL1 and GATA1 occupancy in K562 cells. *B*, shows a table of the SNPs within the +65 CRE. Table columns show the variant ID, *p* value, RAF or risk allele frequency, beta value or odds ratio, phenotypic trait, followed by the first author and Pubmed ID of each respective study. Final column shows the genomic address of the SNP.

To further analyze the sequence bound by LDB1, we analyzed datasets of putative regulatory elements compiled within GeneHancer, an integrated resource of seven different databases (33, 34). The human region syntenic to cluster C scored highly as an elite enhancer within this resource with putative interactions with the *HHEX* TSS (Fig. 3*A*). The region also showed high H3K27 acetylation in hematopoietic cells and occupancy by hematopoietic-specific transcription factors and LDB1 partners, TAL1 and GATA1, in K562 cells (*i.e.* myelo-erythroid leukemia), providing further evidence that the region is a CRE (Fig. 3*A*). We analyzed our data on ChIP-seq analysis from murine Lineage-negative bone marrow progenitors and confirmed occupancy of Ldb1, Tal1, and GATA2 at the murine CRE (personal observation, UPD, PEL, and LL). Furthermore, we found human single nucleotide polymorphisms (SNP) that associated with the phenotypes of lymphocyte count, lymphocyte percentage, neutrophil percentage, and CD20 on B cells (Fig. 3*B*) (35, 36, 37, 38). These associated phenotypes are notable since *Hhex* knockout studies demonstrated its critical role in the function and establishment of common lymphoid progenitor cells and maturation of B cells (11, 12). Interestingly, SNP rs34664889-A associated with lymphocyte counts mutates a GATA site 5′ of the core element from the canonical GATA to AATA. This SNP is also an expression QTL that correlates with reduced *HHEX* expression (beta = −0.111, FDR = 1.14E-5) (39). In summary, the region of LDB1 occupancy contains a conserved core element (Fig. S3) with GATA sites and a composite GATA-E box site, which also shows H3K27 chromatin acetylation and occupancy by two protein partners of the LMO2/LDB1 protein complex. These data mark this region as a *bona fide* CRE and SNPs in the region predict a role for this CRE in human health and disease.

### +65kb enhancer to promoter looping observed in ETP-ALL

**Figure 4:**
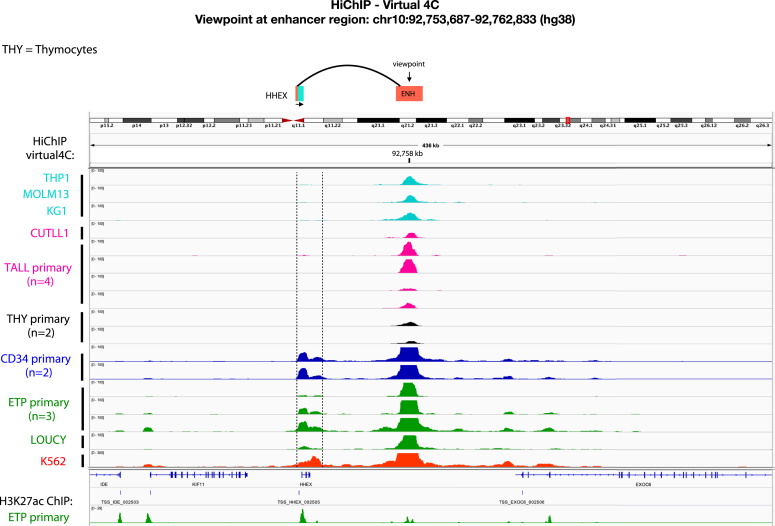
**Virtual 4C analysis shows looping from the +65kb CRE to the proximal promoter/intron one enhancer in human HSPCs and primary human ETP-ALLs.** Genome window of 436kb shows human *HHEX* and surrounding genes. H3K27ac HiChIP data were analyzed by virtual 4C analysis using the ChIP-exo peak sequences in Figure 2, *C* and *D* as the viewpoint anchor. Peaks show regions of cross-linking DNA. Rows from *top* to *bottom* show AML cell line models THP-1, MOLM13, KG1; T-ALL line CUTLL1, followed by four primary T-ALL samples; two primary human thymocyte progenitor cell samples; two primary CD34^+^ HSPC samples; three primary ETP-ALL samples; LOUCY ETP-ALL cell line model; K562 cell line; last row shows ChIP-seq peaks for H3K27ac (acetylation) in a primary ETP-ALL sample. The "H3K27Ac ChIP - ETP primary" track was generated using the HiChIP short reads of the 3 ETP-ALL samples.

LDB1 occupancy at both proximal and distal regulatory elements is observed in beta-globin locus regulation, where distal occupancy at the −20kb locus control region (LCR or super-enhancer) and proximal occupancy at the beta-globin promoter results in looping through LDB1 homodimerization and activation of beta-globin transcription (40, 41, 42). Thus, we tested whether similar looping could occur in the *HHEX* locus. We analyzed a 438 kb region encompassing *HHEX*, the +65kb CRE, and neighboring genes by virtual 4C (chromatin conformation capture) applied to HiChIP analyses from the cell types shown in Figure 4. We used the +65kb CRE as an anchor and interrogated genomic regions for interactions. We observed a single peak over the CRE, consistent with local interactions, in AML cell lines (THP-1, MOLM13, KG1); in *NOTCH1*-driven T-ALL line (CUTLL1); four primary T-ALLs; and, in primary human thymocytes. In contrast, we observed a peak over the *+65kb CRE* and over the *HHEX* proximal promoter/intron one enhancer in primary human HSPC cells (CD34^+^), primary human ETP-ALLs (n = 3), and, in LOUCY (ETP-ALL) and K562 human leukemia cell line models. The observed peaks are evidence of interstitial cross-linking of DNA, confirming looping between the +65kb CRE and the proximal promoter/intron one of *HHEX* in the designated cell types.

## Discussion

In this study, we analyzed one of the most frequent integrations observed in retroviral insertional mutagenesis (RIM) studies. Prior work had attributed distant integrations such as those seen in cluster C to *Hhex* but their functional effect was not clear. Based on multiple genomic datasets, we show that these distal integrations induce *Hhex* upregulation and occur within a CRE that is important in development and leukemogenesis. The ATAC-seq data show chromatin accessibility at cluster C/CRE in the most immature multipotent stem and progenitor cell types (*i.e.* HSPCs) suggesting that this immature stem and progenitor cell type was the target of retroviral integration. Retroviruses are known to integrate into open chromatin (43). These HSPCs were subsequently transformed by the persistent activation of *Hhex*, most likely due to cluster C/CRE looping MLV enhancer elements to the *Hhex* proximal promoter. Other experiments also suggest that the HSPCs were likely targets of retroviral integrations because in bone marrow transduction/transplantation experiments where *Hhex* expression was enforced in HSPCs, T-ALL was induced (44). The MLV integrations near the CRE could create a more potent enhancer at the element. Notably, the MLV integrations do not disrupt the core element described (Fig. 2) so looping may still be required in this posited mechanism. RIM is a major adverse complication of gene therapy using retroviral vectors. Our study shows that intergenic integrations can also be oncogenic and should be monitored for emerging clonality. Prior RIM studies may also be investigated for integrations at potential CRE and super-enhancers, which are important in oncogenesis (27).

The frequent integrations at the cluster C/CRE implicate this site and *Hhex* in oncogenesis but our data annotations show that this element also has a role in normal physiology in both murine and human data. For example, the CRE is an open chromatin region in normal hematopoietic cell populations in a developmental stage-specific pattern based on the ATAC-seq experiments. LDB1 ChIP-exo analysis in human ETP-ALL cells enabled us to narrow the region of LDB1 protein occupancy to 100 to 200 bp (within the +65kb CRE in human genome). It is within this region that we found a composite GATA-E box site that has been described as the preferred binding motif of the LMO2/LDB1 macromolecular protein complex (45, 46). This complex is most likely comprised of two copies of LMO2/LDB1, with LMO2 bound to class I and class II basic helix loop helix heterodimers such as TAL1/E2A and GATA factors through its LIM domains (15). GATA1 and TAL1 peaks are highly enriched at the same core element in K562 cells. All of these subunits of the LMO2/LDB1 complex are master regulators required for the maintenance of HSPCs in normal development and homeostasis. In addition to the E-G-containing core element, the +65kb CRE also has multiple lone GATA sites, which can also bind the LMO2/LDB1 complex. Most strikingly, we found SNVs within lone GATA sites near the core element of the +65kb CRE that were associated with abnormal lymphoid counts or lymphoid proportions. One of these SNPs is a cis-eQTL that affects *HHEX* expression. These human genetic data are remarkable considering that murine knockout studies show *Hhex* has a role in the maintenance and development of the CLP, a key progenitor giving rise to mature B and T cells. Interestingly, *Hhex* knockout mice displayed aberrant recovery from radiation- or chemotherapy-induced bone marrow aplasia implying a functional defect in stem and progenitor cells (11). Perhaps human subjects with the SNPs discussed here may show similar responses to stress hematopoiesis. In summary, the +65kb CRE appears to be a critical element in normal hematolymphoid development that needs further research in both mouse and human models.

One of the most remarkable findings presented in this study is the convergence of data on LMO2 as a driver in ETP-ALL and its relation to *HHEX* activation. Our results and prior work firmly positioned *HHEX* as a downstream target of the LMO2/LDB1 protein complex with the +65kb CRE as the critical element by which *HHEX* activation occurs in ETP-ALL. We attempted deletion of the +65kb CRE in K562 and LOUCY cells by CRISPR/Cas9 using single guide RNAs we but we were unsuccessful. Recent genetic screens suggest that HHEX is essential in ETP-ALL((47)). Thus, if the +65kb CRE is a requisite element for *HHEX* activation, then its deletion would negatively impact cellular growth and would be negatively selected during single cell cloning. The findings of this article support our model for ETP-ALL development, that it is driven by the persistence of a unique transcriptional signature regulated by the LMO2/LDB1 protein complex (48). We and others have described stem cell characteristics conferred upon T-cell progenitor cells by constitutive *Lmo2* expression in mouse transgenic models (48, 49). McCormack, Curtis, and colleagues have shown that many of these properties may also be conferred by enforced expression of Hhex (49). In fact, the *vav-iCre* conditional knockout of *Hhex* markedly attenuated *Lmo2*-induced T-ALL but this was not observed with *Lck-Cre* conditional inactivation (14, 50). The newly identified +65kb CRE may serve as a key point of control, where signals may be integrated for activation of *HHEX* in both normal and malignant physiology. Further study of this CRE will inform our models on T-ALL development and oncogene activation and may give rise to the mechanisms by which oncogene activation may be inhibited to treat ETP-ALL.

## Experimental procedures

### Retroviral integrations

Murine experimentation was approved by the IACUC of the National Cancer Institute. Retroviral insertional mutagenesis and integration site cloning have been previously described (2, 4). Tumor immunophenotyping was performed as described (21). Three T-ALLs analyzed in Figure 1 were isolated from AKXD21.*CD2-Lmo2* (12,315, 12,227) and AKXD21 (12053) mice. Integration sites were initially mapped to mm9 and then on to mm10 using LiftOver (https://genome.ucsc.edu/cgi-bin/hgLiftOver). The mm10 coordinates for the integrations are shown in Table S1 and the bed file can be provided on request. *Hhex* qRT-PCR was done using Taqman probe as previously described (14).

### Genomic datasets

ATAC-seq data were analyzed from the Immunological Genome Project (22). Open Chromatin Regions in the neighborhood of the *Hhex* gene are shown in Fig. S1 and their OCR scores are shown in Table S2 and described in Supplemental text (22). Human T-ALL patient gene expression was analyzed previously (14). ETP-ALL and non-ETP-ALL were compared by *limma* and the gene list is shown in Table S3 (48). Murine T-ALLs were from the B6.*CD2-Lmo2* transgenic model as previously described (14). For differential gene expression analysis, we compared RNA derived from DN3a/b cells from *B6.CD2-Lmo2* v. *Rag2KO* cells (DN3a). We compared this differential gene set with the human dataset by gene set enrichment analysis (GSEA) (51).

### ChIP-exonuclease

Anti-LDB1 ChIP-exonuclease was done using sc-11198 (Santa Cruz Biotechnologies) as described (28). IgG ChIP-exo showed no peaks across the genome. LDB1 ChIP-exo sequencing reads were aligned to hg19 (LOUCY) reference genomes using BWA (52). Genetrack was used to identify LDB1 peaks (53).

DNA within 80 bp of LDB1 ChIP-exo peak summits was analyzed for the presence of the GATA and E-box consensus motifs with zero mismatches using the MEME suite (54). Next, using the GATA or E-box motif location as an anchor, MEME was used to find additional nearby motifs (eg: GATA, E-box, ETS, and RUNX) enriched within 20 bp of the GATA or E-box motif. Bam and Bigwig files showing LDB1 occupancy were visualized in the Washington University Epigenome browser and can be provided upon request (https://epigenomegateway.wustl.edu).

### Virtual 4C analysis

H3K27ac HiChIP-seq was performed as described with the accession numbers for datasets listed in this Methods section (55). HiC-Bench was used to align and filter the data (56). The reads were aligned by bowtie2 (57) and mapped read pairs were filtered by the GenomicTools (58) tools-hic filter command integrated in HiC-bench for known artifacts (57, 59). The filtered reads include multi-mapped reads (‘multihit’), read-pairs with only one mappable read (‘single sided’), duplicated read-pairs (‘ds.duplicate’), low mapping quality reads (MAPQ < 20) and read-pairs resulting from self-ligated fragments. HiC-Bench ‘matrix-sparse’ and ‘virtual4C’ pipelines were used to compute the interactions of each viewpoint in a roll-window fashion as follows (56). For every 200 bp, we summed the valid read pairs in the neighboring ±5 kb region extending up to ±2.5 Mb from the viewpoint. The interactions were CPM normalized by dividing by the total number of valid pairs of the sample and saved in bedgraph format.

## Data availability

All data are contained within this manuscript and supplemental files. Accessions for the genomic datasets are the following: GEO: GSM7798256 for T-ALL_p01; GSM7798257 for T-ALL_p03; GSM7798259 for T-ALL_p06; GSM7798260 for T-ALL_p11; GSM7798258 for ETP-ALL_p04; GSM7798261for ETP-ALL_p15; GSM7798262 for ETP-ALL_p16; GSM7798263 for CD34_2190; GSM7798264 for CD34_2583; GSM7798266 for Thy_2124; GSM7798267 for CUTLL1_DMSO; GSM7798272 for LOUCY-DMSO; SRA: SRR5831491 for K562. Data will also be shared upon request to Dr. Utpal P. Davé, udave@iu.edu.

## Supporting information

This article contains supporting information (14, 22, 60).

## Conflict of interests

The authors declare that they have no conflicts of interest with the contents of this article.

## Supporting information

Table S1

Table S2_Hhex OCRs

Table S4

ETPvsTALL_limma_analysis
