# Supplementary material for: The common murine retroviral integration site activating Hhex marks a distal regulatory enhancer co-opted in human early T-cell precursor leukemia
Source: J Biol Chem. 2025 Jan 27;301(3):108233. doi: 10.1016/j.jbc.2025.108233 (PMC11889976; doi:10.1016/j.jbc.2025.108233)
Supplement: Supplementary text [file mmc5.docx]

**SUPPLEMENTAL TEXT**

**Table S1. Retroviral integrations cloned from AKXD T-cell leukemias and their genomic locations in mm10.** The integrations are listed in this excel file. The Excel columns show the name of the retroviral integration site (RIS), the address of the integration within the mouse genome, distance from the *Hhex* TSS, the AKXD model from which the RIS was cloned, and the sequence of the genomic DNA near the RIS. The integrations are also available as a bed file upon request.

**Figure S1. Open Chromatin Regions at or near *Hhex*.** A, top panel shows a heat map of ATAC-seq reads. Red/yellow denote open chromatin regions and blue/black denote closed chromatin regions based on ATAC-seq performed in the respective hematopoietic stem or progenitor cell; also see Table S2. B, genome window shows where the ATAC-seq reads map with respect to the *Hhex* gene. Hhex_1 and Hhex_2 are the highest ranking putative enhancers identified by Yoshida et al (1).

**Figure S2. *HHEX* mRNA isoform expression in human ETP-ALL.** Similar to the mouse, *HHEX* has two mRNAs emanating from alternate exon 1 transcription start sites, ENST00000472590.6 and ENST00000282728.10. We analyzed RNA-seq datasets for the expression of these 2 isoforms and found the long isoform was the predominant species expressed. TPM values are shown for each isoform for every given cell line or primary T-ALL sample. Reads corresponding to exons are displayed in graphic form on the bottom panels. Expression levels are shown in TPM, transcripts for kilobase million.

**Table S2. ATAC-seq normalized values used to generate heat map in Fig. 1C and Fig. S1.** This file does not convert into a readable format as a pdf and is available upon request. The list of ATAC-seq reads is best displayed in an excel file. ATAC-seq reads are shown as log_2_(value/value_row_mean_) in the excel file. The color scale was generated by splitting the range between the minimum and maximum values into 20 equal bins. The R package s2dv was used to generate a color scale that is also included in Figure 1C. The R scripts are available upon request.

**Table S3. Differential gene expression in human ETP-ALL v. non-ETP-ALL patients.** ETP-ALL differential gene expression analysis has been previously described (2). ETP-ALL and non-ETP cases were classified by immunophenotyping at St. Jude Children’s Research Hospital and differential gene expression was performed by *limma* (3). Columns show from left to right: Affymetrix probe label, coefficient for ETP v. non-ETP, raw P value, fold change, false discovery rate-corrected P value, and gene symbol.

**Table S4. The Lmo2-driven transcriptome in ETP-ALL.** Excel file shows the comparison experiment in Figure 2A-B. *CD2-Lmo2* transgenic DN3 cells were compared with controls to generate a list of Lmo2-driven genes. This list of differentially expressed genes was compared to the ETP v. non-ETP-ALL human differential gene expression list (Table S3) by GSEA using the FGSEA package in R. The list of overlapping genes in shown in this Excel file.

**Figure S3. Core element of LDB1 occupancy aligned to murine and human genomes.** See text for explanation.

**References**

1. Yoshida H, Lareau CA, Ramirez RN, Rose SA, Maier B, Wroblewska A, et al. The cis-regulatory atlas of the mouse immune system. *Cell.* 2019;176(4):897-912. e20.

2. Smith S, Tripathi R, Goodings C, Cleveland S, Mathias E, Hardaway JA, et al. LIM domain only-2 (LMO2) induces T-cell leukemia by two distinct pathways. *PLoS One.* 2014;9(1):e85883.

3. Ritchie ME, Phipson B, Wu D, Hu Y, Law CW, Shi W, et al. limma powers differential expression analyses for RNA-sequencing and microarray studies. *Nucleic Acids Res.* 2015;43(7):e47.
