# Supplementary figures and images for: The common murine retroviral integration site activating Hhex marks a distal regulatory enhancer co-opted in human early T-cell precursor leukemia
Source: J Biol Chem. 2025 Jan 27;301(3):108233. doi: 10.1016/j.jbc.2025.108233 (PMC11889976; doi:10.1016/j.jbc.2025.108233)

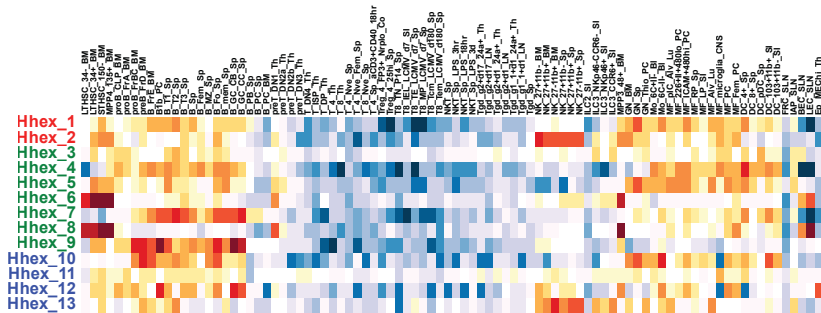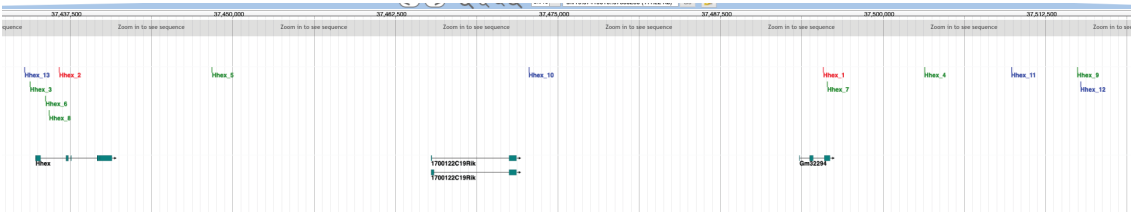

Supplement: Fig S1 [file mmc6.pdf]

Cell-lines

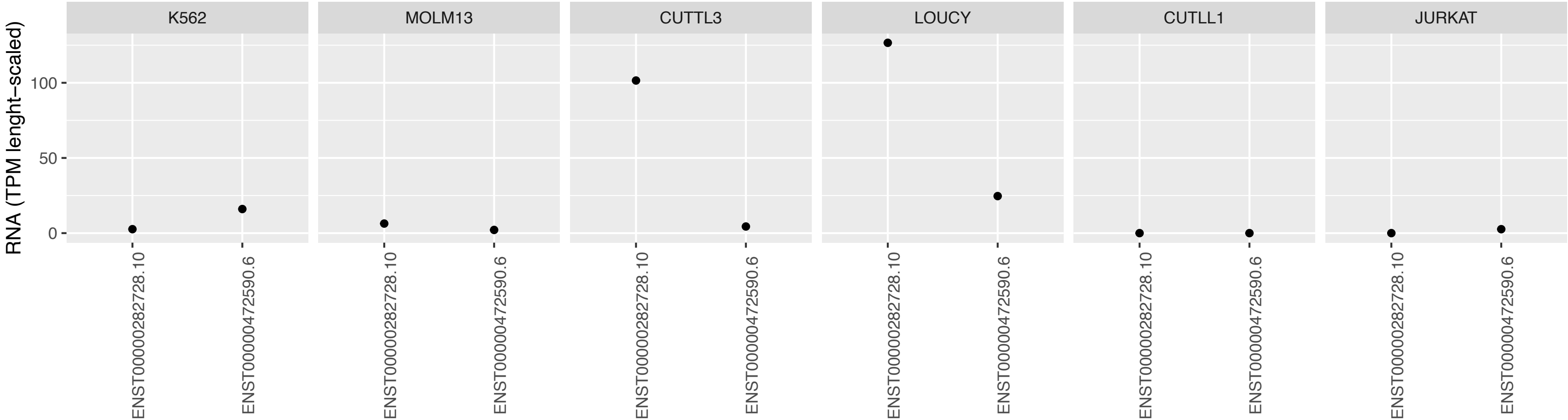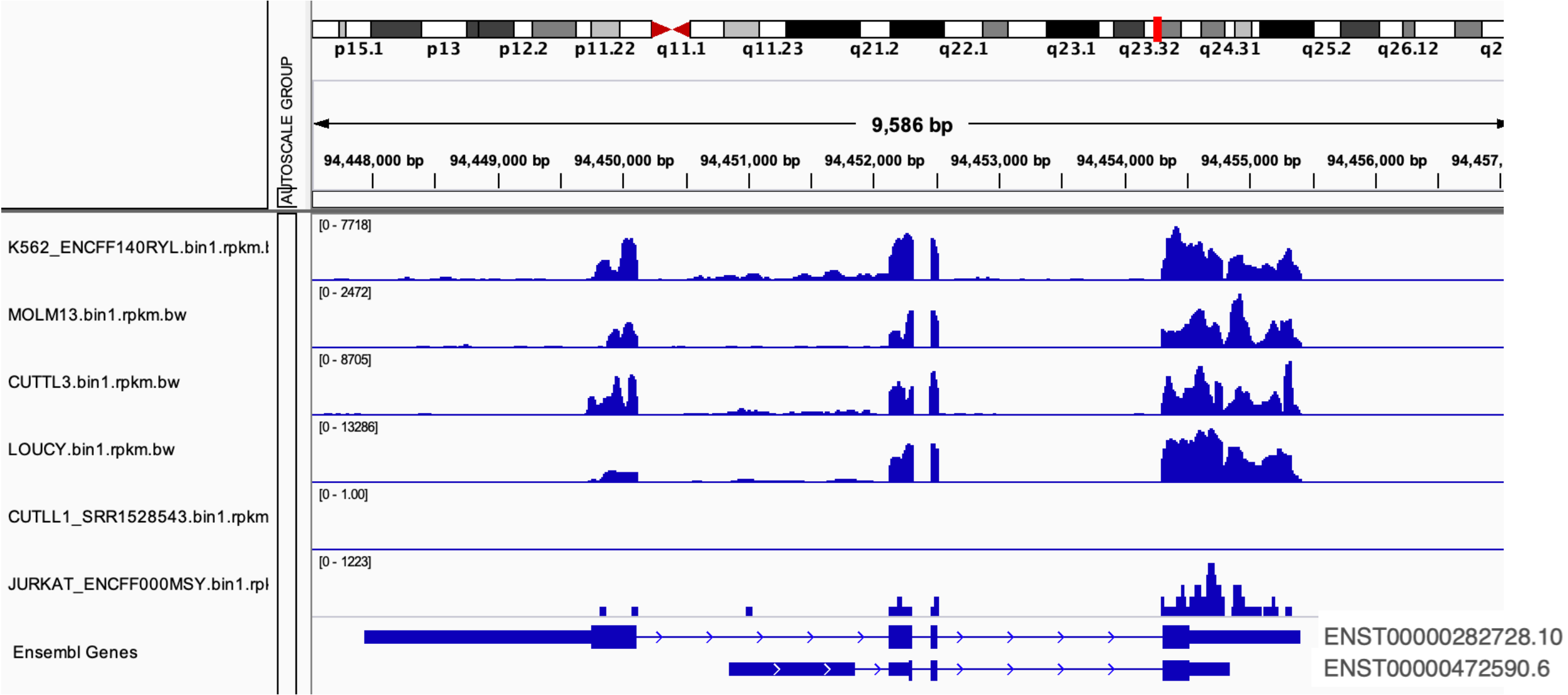

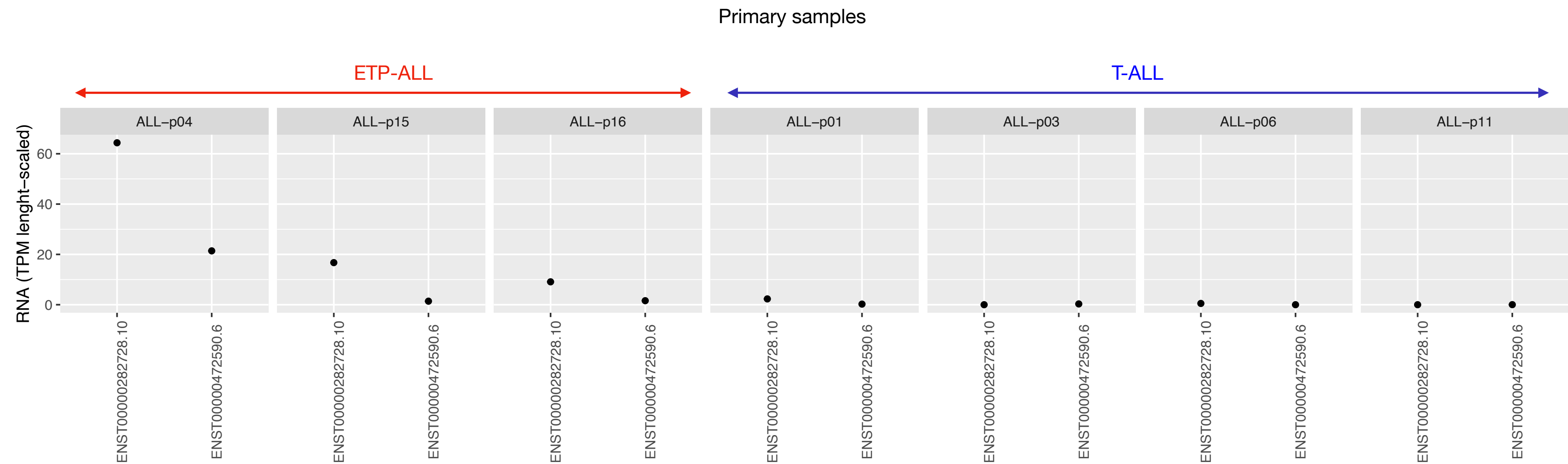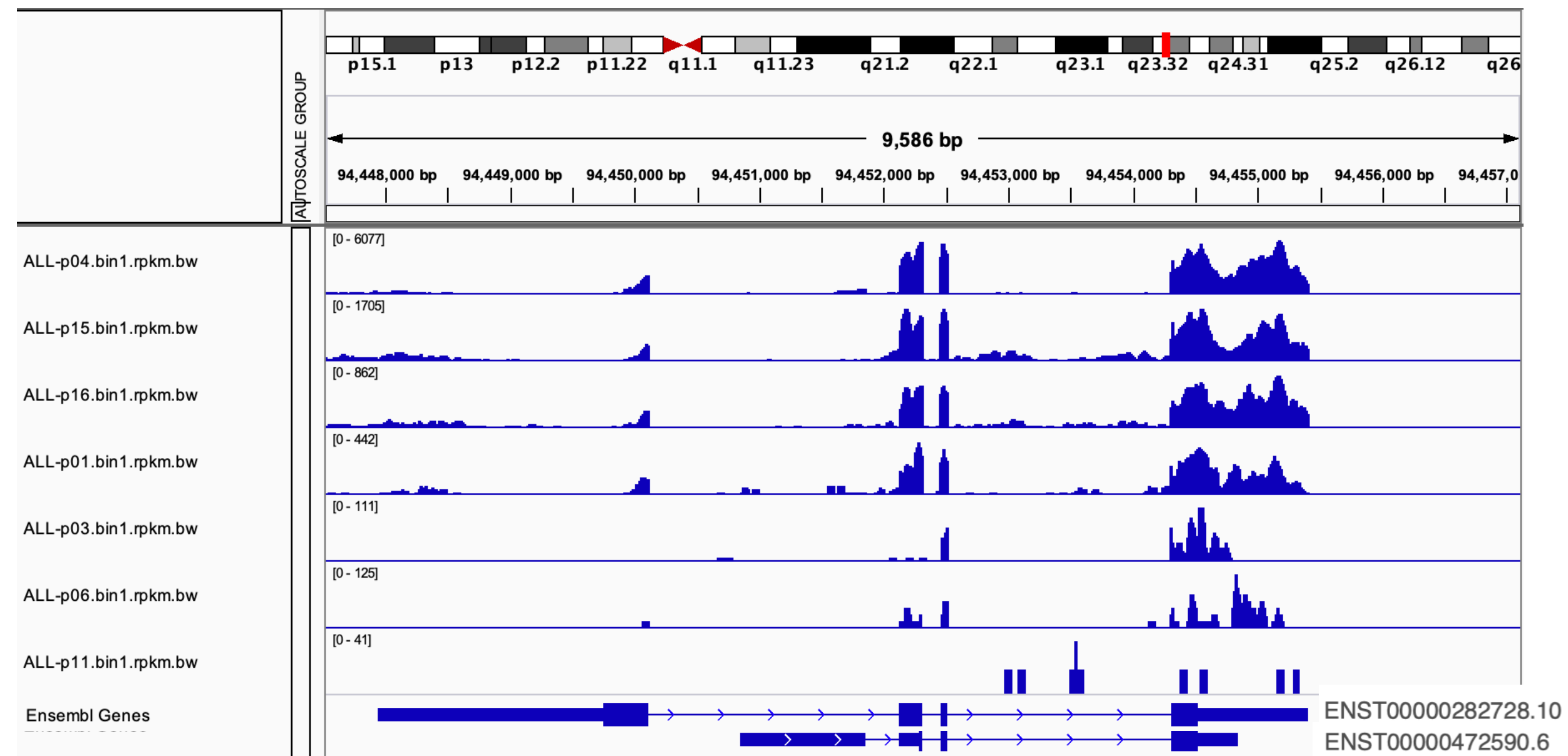

Supplement: Fig S2 [file mmc7.pdf]

# Figure S3

## Mm chr19

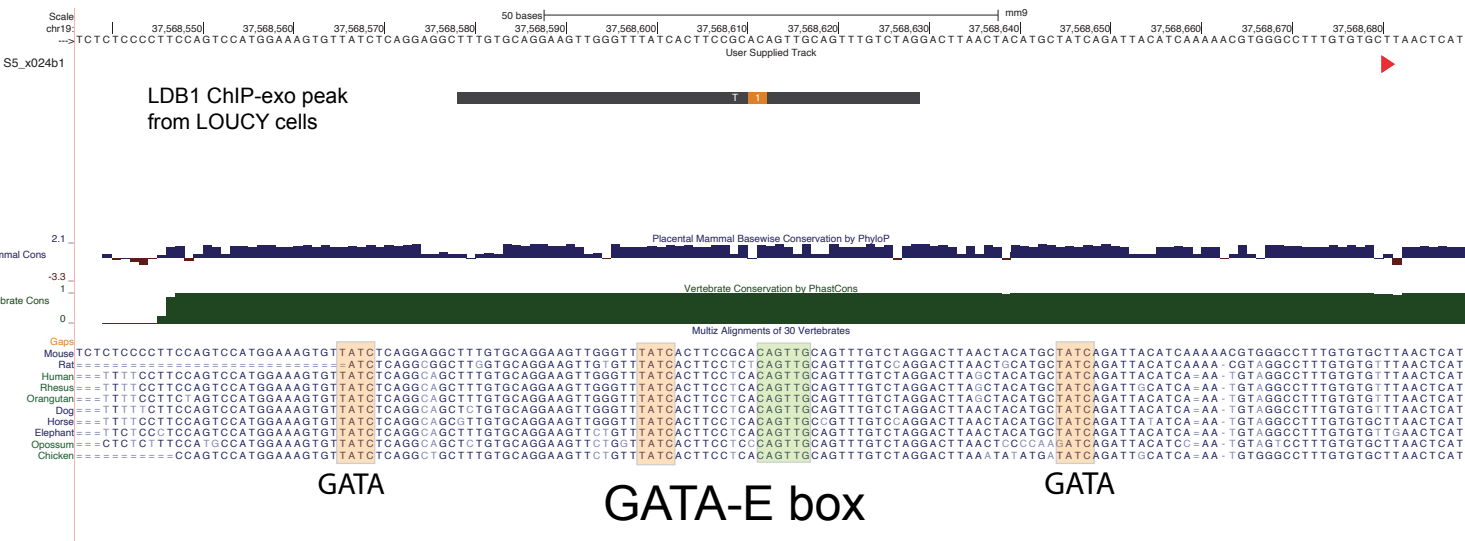

## Hs chr10

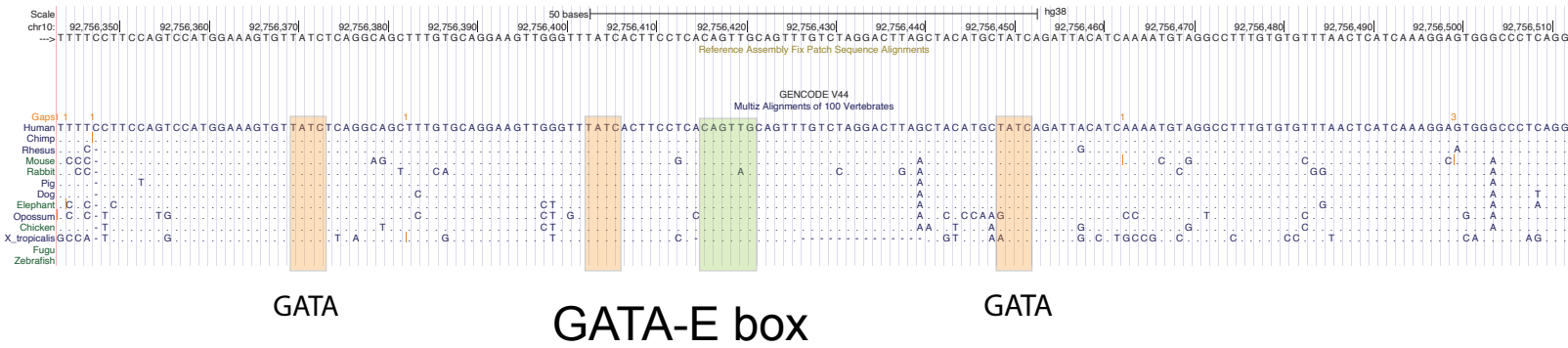

Supplement: Fig S3 [file mmc8.pdf]
